# Autoimmune and Inflammatory Diseases: Identifying New Advances in Immunological Insights

**DOI:** 10.3390/ijms27073085

**Published:** 2026-03-28

**Authors:** Mariapaola Marino, Umberto Basile

**Affiliations:** 1 Dipartimento di Medicina e Chirurgia Traslazionale, Sezione di Patologia Generale, Università Cattolica del Sacro Cuore, L.go F.Vito 1, 00168 Rome, Italy; 2 Fondazione Policlinico Universitario “A. Gemelli” I.R.C.C.S., L.go A. Gemelli 8, 00168 Rome, Italy; 3 Department of Clinical Pathology, Santa Maria Goretti Hospital, 04100 Latina, Italy; u.basile@ausl.latina.it

## 1. Introduction

Dear colleagues,

We launched this Special Issue to explore recent advances in autoimmune and inflammatory diseases, which are associated with a dramatically increasing incidence in high-income countries. The articles that we have collected here provide a broad and multidisciplinary perspective, ranging from pathogenetic mechanisms to potential diagnostic and therapeutic developments. Emerging biomarkers in particular have been recognized, reflecting their key role in disease mechanisms. We are confident that this Special Issue will help to translate scientific insights into improved approaches for the comprehension and management of immune-mediated conditions.

Autoimmune and inflammatory diseases represent an endemic burden to public health due to their severe morbidity. Their treatment is a major challenge in modern medicine, which requires the analysis of the complex interactions between genetic susceptibility, environmental factors, and dysregulated immune responses. Advances in molecular biology, immunology, and clinical research have significantly improved our understanding of the mechanisms underlying immune-mediated tissue damage. In particular, the integration of biochemical pathways, innate immune activation, and cytokine signaling has revealed an intricate network of pathogenic processes involved in the development and progression of many inflammatory and autoimmune disorders [1,2,3].

Innate immunity plays a central role in initiating and amplifying immune responses. Pattern-recognition receptors, including Toll-like receptors (TLRs), detect microbial components as well as endogenous molecules released during cellular stress or damage. The activation of these receptors triggers inflammatory signaling pathways that can promote specific pathways of T cell polarization and in turn provide the final “help” for autoantibody production and chronic immune activation, which is frequently accompanied by increased production of immunoglobulin components [4,5,6,7]. Such mechanisms are particularly relevant in systemic autoimmune diseases such as systemic lupus erythematosus (SLE), where nucleic-acid-sensing pathways contribute to the loss of immunological tolerance and the development of systemic inflammation [8].

Cytokine signaling networks represent another key element in the regulation of immune responses. Pro-inflammatory mediators such as TNF-α, IL-1, and IL-6 orchestrate inflammatory reactions in different tissues and organs, while anti-inflammatory cytokines such as TGF-β and IL-10 may exert regulatory functions depending on the immunological context [9,10,11,12]. These mediators participate in both chronic autoimmune diseases and acute inflammatory conditions, contributing to immune cell activation, tissue injury, and systemic inflammatory responses.

Alongside mechanistic insights, significant efforts have been directed toward identifying reliable biomarkers capable of improving diagnosis, predicting disease activity, and guiding therapeutic decisions. Advances in molecular technologies, including proteomic and immunological approaches, are enabling the identification of disease-specific immune signatures and circulating biomarkers associated with autoimmune disorders and chronic inflammatory diseases [13,14,15,16].

The collection of articles in this Special Issue contributes to this growing body of knowledge by exploring and updating different aspects of immune dysregulation, including innate immune signaling, mitochondrial dysfunction, cytokine networks, and biomarker discovery across several autoimmune and inflammatory diseases.

## 2. Overview of the Articles

The studies collected in this Special Issue are of a wide scope of research, providing multidisciplinary perspectives on immune-mediated diseases and emphasizing both pathogenetic mechanisms and potential clinical applications. Several articles focus on systemic autoimmune diseases, particularly systemic lupus erythematosus.

Cytokine signaling pathways are extensively discussed across several contributions. Given IL-10’s dual immunoregulatory and pro-inflammatory functions, Richter and coworkers (contribution 1) evaluate its role in systemic lupus erythematosus and examine its association with inflammatory markers and autoantibody production, showing a significant positive correlation between IL-10 and IL-6, anti-nuclear antibodies (ANA), and C-reactive protein (CRP) levels. Although IL-10 is generally regarded as an anti-inflammatory cytokine, the findings suggest that its levels may reflect a broader immune activation rather than directly correlating with clinical disease activity. No correlations were found between IL-10 levels and disease activity, specific organ involvement, or current treatment regimens. This observation strengthens the complex and context-dependent functions of cytokines in autoimmune and inflammatory diseases where regulatory and pro-inflammatory mechanisms may coexist, as previously reported in different settings [12,17,18].

Biomarker discovery represents another major theme within the collection. The second text published in this Special Issue by Lee et al. (contribution 2) identifies anti-chaperonin-containing t-complex polypeptide-1 (TCP1) antibody as a novel autoantibody associated with SLE through proteomic screening techniques. These findings illustrate the increasing relevance of using protein chip microarrays as an excellent experimental technique for identifying interactions between proteins, enzyme–substrate interactions, antibody–antigen reactions, and identifying disease-specific biomarkers and improving diagnostic accuracy in complex autoimmune conditions. Subsequent validation analyses reveal that anti-TCP1 antibody is significantly elevated in patients with SLE compared with healthy controls and individuals affected by other autoimmune diseases.

The article by Xu et al. (contribution 3) explores the relationship between mitochondrial dysfunction and autoimmunity in SLE using experimental genetic model knockout mice lacking Fam210b, which is a mitochondrial protein that has been shown via bioinformatics to be a key regulator. The study demonstrates that alterations in mitochondrial regulation may lead to increased production of reactive oxygen species and abnormal immune activation. Oxidative stress appears to influence immune cell differentiation and contribute to the development of autoimmune phenotypes. These observations align with growing evidence that mitochondrial metabolism and oxidative stress represent important regulators of immune function and inflammation [19,20].

Cerrillos-Gutiérrez’s article (contribution 4) examines the involvement of nucleic-acid-sensing pathways in lupus nephritis by analyzing the expression of TLR7 and TLR9 associated with innate immune activation. The results suggest that the altered activation of these pathways may contribute to renal inflammation and disease progression, supporting the concept that dysregulated innate immunity plays a crucial role in lupus pathogenesis. These findings are consistent with previous evidence indicating that endogenous nucleic acids released from damaged cells can activate innate immune receptors and trigger sustained inflammatory responses [1,4].

Additional reviews and updates in this Special Issue have extended the discussion to autoimmune diseases affecting other organs.

Advances in biomarker research for demyelinating diseases of the central nervous system are reviewed by Florea and coworkers (contribution 5), highlighting the potential of molecular markers to improve diagnostic precision and support personalized therapeutic strategies.

Acute pancreatitis (AP) displays a severe evolution, and is seeing an increased incidence in adults and children. Mititelu (contribution 6) examines the role of elevated IL-6 levels during the initial phases of pancreatic inflammation in association with systemic inflammatory responses and more severe disease outcomes. IL-6 plays a central role in the acute-phase response; thanks to its two distinct receptor pathways acting in the different cell types (classical and trans-signaling), it may drive transition to and monocyte-infiltration into inflamed tissue [21] and stimulate hepatic production of inflammatory mediators [22]. The authors identified IL-6 as a useful indicator for early risk stratification in inflammatory conditions such as AP.

In addition to AP, inflammatory mechanisms involved in other organ-specific disorders were analyzed. The review by Pocino et al. (contribution 7) explores the diverse biological functions of TNF-α in skin physiology and pathology. TNF-α regulates multiple cellular processes, including immune cell recruitment, apoptosis, and angiogenesis, and its dysregulation has been implicated in several dermatological conditions such as psoriasis and inflammatory skin diseases. The therapeutic success of TNF-α inhibitors further underscores the importance of cytokine signaling pathways in chronic inflammatory disorders [9].

Autoimmune thyroid disorders are reviewed by Tywanek (contribution 8), with particular attention to emerging diagnostic markers and the immunological processes responsible for thyroid-specific autoantibody production.

Inflammasome-mediated inflammation and IL-1 signaling are also addressed in the context of hereditary autoinflammatory syndromes. These disorders are characterized by dysregulated innate immune responses leading to excessive IL-1 production. The ninth contribution to this Special Issue is a commentary by Rigante (contribution 9) showing how the long-term blockade of IL-1 has been found to restore the clinical equilibrium in systemic inflammasomopathies of childhood, with a well-documented safety profile, significantly improved clinical outcomes and valuable insights into the fundamental role of innate immune pathways in inflammatory diseases [10].

Finally, neurological autoimmunity is studied through a clinical report by Števková and coworkers (contribution 10), describing the complex case of anti-NMDA receptor encephalitis associated with an ovarian teratoma. This case illustrates the mechanisms of paraneoplastic autoimmunity and antibody-mediated neuronal dysfunction, providing us with key information for treatment: a failure to standardize first- and second-line immunotherapy requires early, aggressive therapy with an intrathecal application of methotrexate in order to prevent poor long-term patient outcomes.

## 3. Conclusions

Collectively, the studies in this Special Issue highlight the rapid progress made in understanding autoimmune and inflammatory diseases from a molecular and immunological perspective. Several key concepts emerge from this body of research.

First, innate immune pathways are important in initiating and sustaining autoimmune responses. Mechanisms involving pattern-recognition receptors, inflammasome activation, mitochondrial dysfunction, and oxidative stress contribute to immune dysregulation and represent promising targets for future therapeutic interventions.

Second, cytokine networks remain central regulators of inflammatory processes. Mediators such as TNF-α, IL-1, IL-6, and IL-10 orchestrate complex interactions between immune cells and tissues, influencing both local inflammation and systemic immune responses. Their roles as biomarkers and therapeutic targets continue to be actively explored in multiple inflammatory and autoimmune diseases.

Finally, advances in biomarker discovery and molecular diagnostics are directing us toward more precise approaches for disease classification and management. The integration of biochemical research, immunological insights, and clinical investigation is essential for translating these discoveries into improved diagnostic tools and more effective therapeutic strategies.

## Data Availability

Not applicable.
